# Evaluation of the Antibody in Lymphocyte Supernatant Assay to Detect Active Tuberculosis

**DOI:** 10.1371/journal.pone.0169118

**Published:** 2017-01-13

**Authors:** Margaretha Sariko, Caitlin Anderson, Buliga S. Mujaga, Jean Gratz, Stellah G. Mpagama, Scott Heysell, Gibson Kibiki, Blandina Mmbaga, Eric Houpt, Tania Thomas

**Affiliations:** 1 Kilimanjaro Clinical Research Institute, Moshi, Tanzania; 2 Kilimanjaro Christian Medical University College, Moshi Tanzania; 3 Kilimanjaro Christian Medical Centre, Moshi, Tanzania; 4 University of Washington, Seattle, United States of America; 5 University of Virginia, Charlottesville, United States of America; 6 Kibong’oto Infectious Diseases Hospital, Kilimanjaro, Tanzania; Colorado State University, UNITED STATES

## Abstract

**Background:**

We aimed to evaluate the antibody in lymphocyte supernatant (ALS) assay as a biomarker to diagnose tuberculosis among adults from Tanzania with and without HIV.

**Methods:**

Adults admitted with suspicion for tuberculosis had sputa obtained for GeneXpert MTB/RIF, acid-fast bacilli smear and mycobacterial culture; blood was obtained prior to treatment initiation and after 4 weeks. Adults hospitalized with non-infectious conditions served as controls. Peripheral blood mononuclear cells were cultured unstimulated for 72 hours. Anti-mycobacterial antibodies were measured from culture supernatants by ELISA, using BCG vaccine as the coating antigen. Median ALS responses were compared between cases and controls at baseline and between cases over time.

**Results:**

Of 97 TB cases, 85 were microbiologically confirmed and 12 were clinically diagnosed. Median ALS responses from TB cases (0.366 OD from confirmed cases and 0.285 from clinical cases) were higher compared to controls (0.085, p<0.001). ALS responses did not differ based on HIV status, CD4 count or sputum smear status. Over time, the median ALS values declined significantly (0.357 at baseline; 0.198 after 4-weeks, p<0.001).

**Conclusions:**

Robust ALS responses were mounted by patients with TB regardless of HIV status, CD4 count, or low sputum bacillary burden, potentially conferring a unique niche for this immunologic biomarker for TB.

## Introduction

Tuberculosis (TB) continues to be leading driver of global morbidity and mortality, accounting for over 1.5 million deaths annually. Tanzania represents one of the World Health Organization’s (WHO) top-20 high-burden countries for TB, with an annual incidence of 327 (155–561)/100,000 population. Although the rollout of the GeneXpert MTB/RIF (Cepheid, Sunnyvale, USA) has improved access to rapid diagnostics, undiagnosed cases persist, diagnostic delays complicate treatment outcomes, and there are still considerable gaps to fill in order to achieve WHO’s ambitious global targets to “End TB” [1, 2].

The majority of diagnostic tests for TB rely on the use of sputum specimens; however, issues such as inadequate volume, excessive saliva, and paucibacillary or advanced disease states often limit specimen quality and subsequent diagnostic yield. A biomarker for TB that relies on non-respiratory specimens would be an advance to the field, and one that retains accuracy in HIV-positive individuals is especially needed [3]. The Antibody in Lymphocyte Supernatant (ALS) assay may represent one such biomarker based on promising results from adults from Bangladesh with TB, and from Ethiopia with TB/HIV co-infection [4–6].

The principles behind the ALS assay rely upon the activity of pathogen-specific antibody-secreting cells: during active infection, B-cell activation triggers antibody secreting cells, or immature plasma cells, which transiently migrate through the bloodstream while en route to the site of infection [7, 8]. For TB, the ALS assay captures the spontaneous release of anti-mycobacterial IgG antibodies from peripheral blood and has been used for the diagnosis of active TB and treatment monitoring [4, 9, 10]. Although this is an antibody-based assay, it differs from traditional serologic tests by discarding the serum and measuring incident antibody production directly from the lymphocytes. Thus, it can serve as a biomarker of immune response to TB antigens that are present during active disease but not latent infection [5]. In this study, we aim to evaluate the performance of the ALS assay in diagnosing active TB and monitoring response to TB treatment among adults with and without HIV from Tanzania.

## Materials and Methods

### Study design and population

This is a case control study involving two sites, Kibong’oto Infectious Disease Hospital (KIDH) and Kilimanjaro Christian Medical Centre Hospital. KIDH is the national referral hospital for TB. Between January 2014 and May 2015, adults admitted to KIDH with concern for pulmonary TB were referred to the study team. Patients were recruited if they were >18 years of age and were imminently starting treatment for pulmonary TB. Exclusion criteria included: hemoglobin level <7.0 g/dL, Karnofsky score <50, or inability/unwillingness to provide informed consent. Eligible participants underwent a standardized medical interview and anthropometrics. Evaluation for TB included clinical symptom review, chest radiograph, and microbiologic analysis of sputum; tuberculin skin testing is not part of the routine evaluation as per national guidelines [11]. Tanzania performs routine BCG vaccination at birth with an estimated coverage of 95% [12].

At the time of TB treatment initiation, a blood sample and overnight pooled sputum sample were collected as previously described [13]. A repeat assessment was conducted after 4 weeks. Sputum specimens were transported to the Mycobacteriology Laboratory at Kilimanjaro Clinical Research Institute for processing within 8 hours, where testing included GeneXpert MTB/RIF (Cepheid), concentrated acid-fast bacilli (AFB) smear, and mycobacterial culture (Mycobacterial Growth Indicator Tubes, MGIT and Löwenstein-Jensen solid culture media, Becton, Dickinson and Company, Franklin Lakes, USA)[14]. Cultured isolates were confirmed as *Mycobacterium tuberculosis* (Mtb) complex by DNA probe (Gen-Probe, USA). Drug sensitivity testing was performed using the MGIT 960 system (BD and Co).

Participants were classified as “confirmed TB cases” if they had Mtb detected by Xpert MTB/RIF and/or TB culture. Participants who did not have microbiologic confirmation, but were still being treated for TB, were classified as “clinically diagnosed TB cases.” Xpert MTB/RIF results and/or drug sensitivity testing results were also used to characterize cases as having drug-susceptible or drug-resistant TB. All cases were treated with first- or second- line anti-TB drugs according to their drug-sensitivity profiles and national guidelines, as per the treating physicians [11, 15]. All patients were tested for HIV if not already being treated with antiretrovirals; HIV-positive patients were initiated on antiretrovirals within 8 weeks of starting TB treatment.

Between April and July 2014, adults who were admitted to Kilimanjaro Christian Medical Centre Hospital for non-infectious conditions, such as hypertension, diabetes, anemia/gastrointestinal bleeding, and abdominal pain, were recruited as hospitalized controls. Patients were eligible if they were >18 years of age and provided informed consent. Exclusion criteria included current receipt of TB treatment, a prior history of TB, or presence of signs or symptoms that could be consistent with TB. Attempts were made to match controls to cases based on age (within 5 years). Participants underwent a brief standardized medical interview; clinical and anthropometric data were collected. A blood sample was collected at the time of enrollment; tuberculin skin testing was not performed.

### Antibodies from lymphocyte supernatant

The ALS assay was conducted as previously described [4]. Briefly, approximately 8ml of blood was collected in vacutainers pre-loaded with ficoll (CPT vacutainers, BD and Co.) and transported for processing within 3 hours according to the manufacturer's instructions [16]. The peripheral blood mononuclear cell (PBMC) suspension was isolated and cells were washed. Viable cells were counted, with yields consistently >97%. Cells were re-suspended in phosphate buffer saline at a concentration of 5x10^6^ cells/mL. Using 96-well tissue culture plates, 200μl of the re-suspended cells were cultured without antigenic stimulation in RPMI with 10% fetal bovine serum (GIBCO, Carlsbad, California, USA), 2 mM l-glutamine and a 1% concentration of an amphotericin B-penicillin-streptomycin mixture (Sigma-Aldrich, St. Louis, Missouri, USA) in 5% CO_2_ bags at 35°C for 72 hours. Cell supernatants were collected and stored with protease inhibitors at -80°C for batch analysis.

Freeze-dried glutamate-Bacille Calmette Guerin vaccine (BCG, Japan BCG Laboratories, Tokyo, Japan) was used as the coating antigen. Microtiter plates (Nunc Maxisorp, Sigma-Aldrich) were coated with BCG (10μg/well in carbonate buffer, pH 9.8) and washed after overnight incubation at 4°C. Bovine Serum Albumin (1.5% BSA) in phosphate-buffered saline (pH 7.2) was used to block non-specific antibodies.

Lymphocyte supernatants were thawed; 100μL was added to duplicate wells along with controls and the plates were incubated at 37°C. Positive controls consisted of pooled serum from Tanzanian adult patients with culture-confirmed TB. Negative controls consisted of PBS blanks and RPMI tissue culture media. After washing, the plates were incubated with rabbit anti-human IgG-horseradish peroxidase conjugate. *O*-phenylenediamine was used as a developer and 2M sulphuric acid was used to stop the reaction. Absorbance was measured at 492 nm optic density (OD). ALS responses are reported as the normalized value after subtracting the blanks. All ELISAs were performed in duplicate; the coefficient of variation was 6.4% between duplicates.

### Ethical approval

Ethical clearance was obtained from the National Institute of Medical Research, Kilimanjaro Christian Medical University College ethical committee, and University of Virginia. Eligible patients underwent the informed consent process; written informed consent was obtained from all enrollees.

### Data analysis

Descriptive analyses, simple frequencies, and distribution of results were evaluated. ALS responses between cases (microbiologically confirmed or clinically diagnosed) and controls were compared using the Kruskal-Wallis test for non-parametric data. Due to outliers in each of the “case” categories, regression analysis was used to evaluate characteristics that could be associated with positive ALS result, including age, gender, smoking status, alcohol use, body mass index, diabetes mellitus status, HIV status, prior history of TB, drug-susceptibility profile results. Receiver operator characteristic (ROC) analyses were performed to assess the optimal sensitivity and specificity of the ALS assay based on the threshold for positivity. Among TB cases, serial ALS responses were compared using the Wilcoxon signed rank test. The change in ALS titers over time was evaluated to determine any association with favorable TB treatment outcome (cure or treatment completed) using univariate and multivariate regression analyses. Data were tabulated and analyzed using Statistical Packages for Social Science (SPSS) version 22; a p-value <0.05 was considered significant.

## Results

### Participant characteristics

A total of 125 patients were enrolled, including 97 participants with TB and 28 hospitalized controls without TB. Of the 97 TB cases, 85 (88%) had a microbiologic confirmation, while 12 (12%) were diagnosed on clinical grounds. The demographic and clinical characteristics of all participants can be seen in Table 1. Cases and controls differed by gender, however this did not affect ALS responses in the regression model (data not shown). Among the 22 HIV/TB co-infected participants, 17 (68%) had been initiated on HIV therapy prior to admission, and the median CD4 count was 116 cells/mm^3^ (IQR: 36–236).

**Table 1:** Demographic and clinical characteristics.

Characteristics	Confirmed TB,	Clinically diagnosed TB,	Controls,
n, (%)	n = 85	n = 12	n = 28
**Age**, mean years ± SD	40 ±14	40 ±9	42 ±15
**Gender**			
Male	68 (80)	7 (58)	7 (25)
Female	17 (20)	5 (42)	21 (75)
**Domicile**			
Northern Zone	56 (66)	1 (8)	22 (79)
Central Zone	18 (21)	0 (0)	0 (0)
Southern Zone	1 (1)	0 (0)	0 (0)
Lake Zone	3 (4)	4 (33)	0 (0)
Eastern Zone	7 (8)	7 (59)	0 (0)
Unknown			6 (21)
**Current or prior smoking**	16 (19)	1 (8)	2 (7)*
**Current or prior alcohol use**	36 (42)	3 (25)	10 (36)*
**Known diabetes mellitus**	3 (3)	0 (0)	3 (11)
**HIV infected**	18 (21)	6 (50)	4 (14)*
On anti-retroviral therapy (n, % of HIV infected)	12 (67)	5 (83)	n/a
CD4^+^ T-cell count (median cells/mm^3^, range)	132 (39–239)	44 (14–260)	n/a
**Body Mass Index**			
Normal (BMI: 18.5–24.9)	55 (65)	5 (42)	22 (79)
Mildly malnourished (BMI: 17–18.5)	16 (19)	3 (25)	
Moderately malnourished (BMI: 16–16.9)	8 (9)	1 (8)	
Severely malnourished (BMI: <16)	6 (7)	3 (25)	
Unknown			6 (21)
**Current TB type**			
Drug susceptible TB	55 (65)	n/a	n/a
MDR TB	30 (35)		
**Prior history of TB**	39 (46)	6 (50)	0 (0)
**Number of episodes** (n, % with prior TB)			
1 Episode	24 (62)	2 (33)	n/a
> 2 Episodes	15 (38)	4 (67)	
**TB treatment outcome**			n/a
Cured/completed	54 (64%)	4 (33%)	
Died/failed	9 (11%)	3 (25%)	
Other^ǂ^	22 (26%)	5 (42%)	

*six had unknown status.

^ǂ^ including default, transfer, or otherwise unknown.

### Evaluation of ALS as a diagnostic biomarker

**Fig 1:**
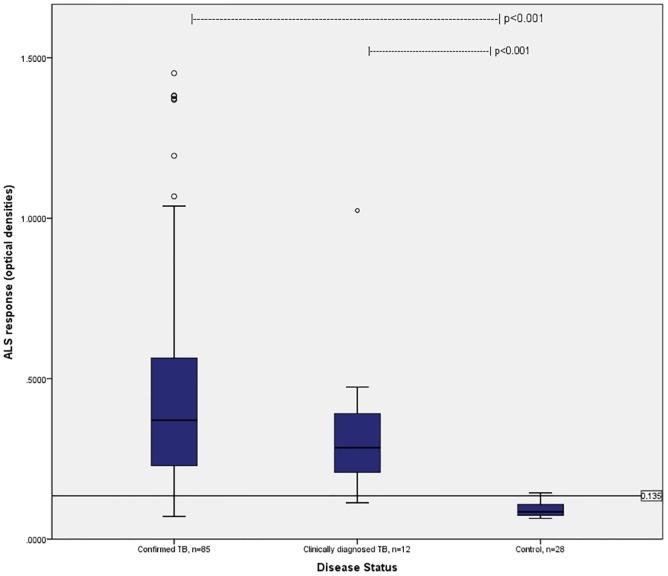
ALS response by disease status. Data are shows as median values and interquartile range within the boxplots, and range within the whiskers. Compared to controls, ALS responses were higher among both TB groups, p<0.001 by Kruskal-Wallis test.

The median ALS responses among confirmed TB cases (0.366 OD, IQR: 0.288–0.564) and clinically diagnosed TB cases (0.285 OD, IQR: 0.188–0.411) were higher than those from controls (0.085 OD, IQR: 0.073–0.110, p <0.001, Fig 1). After controlling for other characteristics, only the ALS responses from “confirmed TB” cases remained significantly different compared to those from controls; these results were not affected by removal of the outliers.

Importantly, ALS responses among TB cases were not diminished in HIV-positive individuals (median ALS response of 0.302 OD [IQR: 0.194–0.562] among the 24 HIV-positive individuals versus 0.362 OD [IQR: 0.245–0.546] among the 73 HIV-negative individuals, p = NS). Within the HIV-positive subset, ALS responses did not correlate with CD4^+^ T-cell count (Pearson’s r = 0.103, p = NS). Further analyses based on use of antiretroviral therapy were not possible due to small sample sizes in the subgroups. Additionally, within the entire cohort, ALS response did not differ based upon baseline smear status (positive or negative).

**Fig 2:**
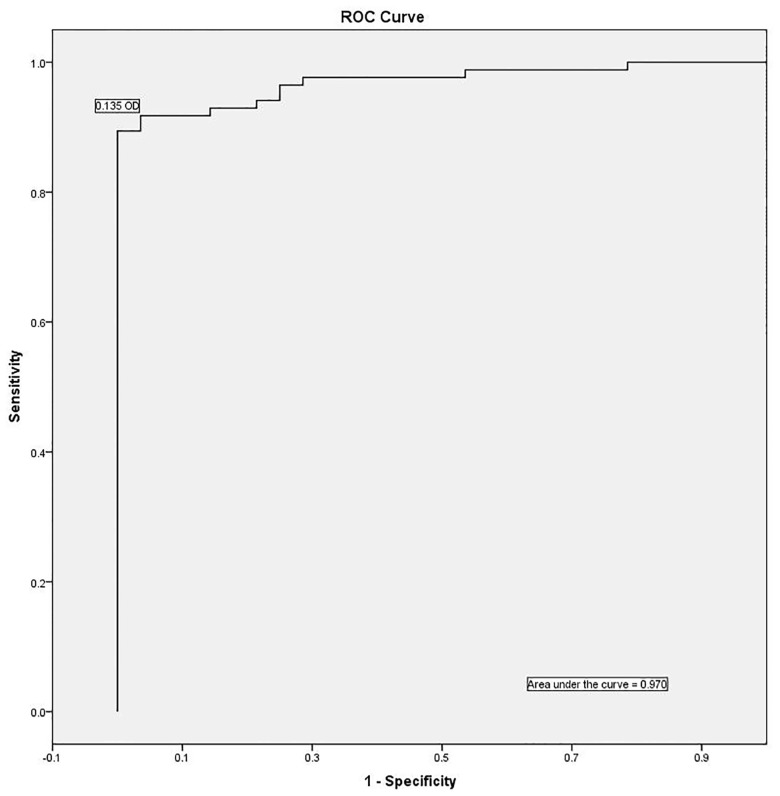
Receiver operator characteristic curve. Receiver operator characteristic (ROC) curves were created using ALS responses from microbiologically-confirmed TB cases (n = 85) and non-TB controls (n = 28). The area under the curve is 0.970. The arrow indicates a proposed threshold for assay positivity which maximizes sensitivity (92%) and specificity (96%).

Receiver operator characteristic curves were created by comparing ALS responses from microbiologically confirmed TB cases and controls, see Fig 2. Accuracy for diagnosis of TB was high with an area under the curve of 0.970. The optimal threshold for positivity, 0.135 OD, would provide 92% sensitivity and 96% specificity.

### Evaluation of ALS as a response-predictive biomarker

**Fig 3:**
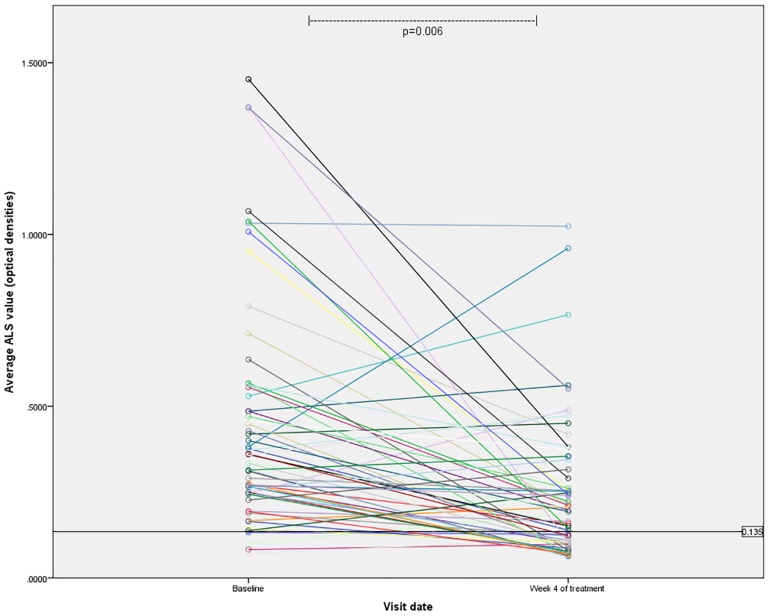
ALS results over time. Individual ALS responses for 61 TB cases are displayed over two time points during TB treatment. The median response at baseline was 0.357 (IQR: 0.247–0.559) and after four weeks of TB treatment, the median response declined significantly to 0.198 (IQR: 0.102–0.349, p = 0.006 by Wilcoxon signed rank test).

**Fig 4:**
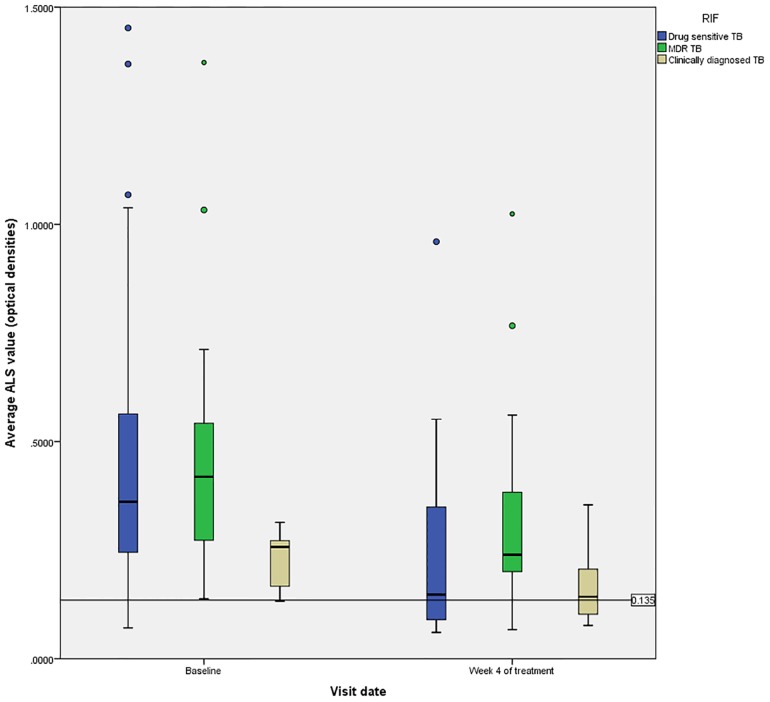
Reduction in ALS responses over time, based on drug resistance profile. Median ALS responses are displayed at two different time points by drug resistance profile. After four weeks of TB treatment, adults with drug-sensitive TB demonstrated a greater reduction in ALS responses, although this was not statistically significant (p = 0.057, Wilcoxon signed rank test).

Follow up blood specimens four weeks after anti-TB therapy initiation were available from 61 TB cases (63%), which included 36 (59%) with drug-sensitive TB, 19 (31%) with MDR-TB, and 6 (10%) with clinically diagnosed TB. Fig 3 demonstrates the reduction in ALS responses over time. Overall, the median values declined significantly between the measurements at baseline (0.357, IQR: 0.247–0.559) and 4 weeks post treatment initiation (0.198, IQR: 0.102–0.349, p<0.001). When examining the reduction in ALS responses based on type of TB (drug-sensitive, MDR, or clinically diagnosed), there was a trend towards a having a greater reduction among those with drug-sensitive TB (p = 0.057, Fig 4). The magnitude of decline in ALS responses over time was not associated with TB treatment outcome by univariate or multivariate regression analysis.

## Discussion

In this cohort of adults from Tanzania, those with microbiologically confirmed TB had significantly higher ALS responses at the time of TB diagnosis compared to controls who were hospitalized for non-infectious causes. Robust ALS responses were mounted regardless of HIV status, CD4 count, or low sputum bacillary burden. These findings support our hypotheses that immune activation from Mtb can be detected through incident secretion of anti-BCG immunoglobulins from plasmablasts found in PBMCs.

In our longitudinal assessment of ALS measurement, we noted a significant reduction in ALS responses after the four weeks of TB therapy. These declines were most pronounced among those confirmed to have drug-sensitive TB, which suggests that potent anti-TB therapy may be associated with reduced TB antigenic stimulation. These trends are similar to those previously demonstrated among adults and children [10, 17]. During the time interval tested in our protocol, the reductions in ALS responses were not associated with treatment outcome. However, the evaluation of ALS as a biomarker predictive of early relapse may be relevant for studies that evaluate shorter courses of TB treatment [18–21]. Therefore, further examination including multiple follow up time points among a larger cohort may be warranted to evaluate the utility of this biomarker in predicting treatment response and outcome.

Our finding that HIV status, including patients with considerable T-cell immunosuppression, did not affect the ability to mount an equivalent ALS response is notable. Similar findings have been seen by Ashenafi et al in their cohort of 84 adults from Ethiopia with TB, including 27 with TB/HIV co-infection. Despite the majority of co-infected participants having CD4^+^ T-cell counts <200 cells/mm^3^, they also found that ALS responses did not differ significantly between those with HIV/TB compared to those with TB alone [5]. In conjunction these results affirm that this assay holds potential to fill a gap in the current biomarker landscape.

Biomarkers for TB are greatly needed to improve clinical practice and to hasten drug development research, especially among HIV/TB co-infected populations [21]. Our work suggests that the ALS assay could serve as a diagnostic and response-predictive biomarker among adults by harnessing B-cell stimulation to gauge TB disease activity. There is growing evidence to suggest that B-cells mediate protection for TB through antigen presentation, cytokine production, and antibody production via interactions with cell mediated immunity [22–25]. When immunologic biomarkers are dependent on T-cell immunity, as is the case for the currently available tuberculin skin test and interferon-γ release assays, concern is raised about reduced performance among populations with concomitant HIV co-infection or other immunocompromising conditions [26]. The ALS assay may work well in co-infected patients by leveraging the state of B-cell dysregulation that is known to occur with HIV, which includes hyperactivity of plasmablasts [27, 28]. In HIV infection, circulating plasmablasts are found to rise early and maintain high levels in viremic states; however, the majority of plasmablasts are not HIV-specific and likely reflect non-specific immune activation [29, 30]. We did not detect elevated ALS response among the four HIV-positive hospitalized controls (range 0.065–0.127). It is possible that the magnitude of anti-BCG IgGs we detected from co-infected individuals stem from an increased overall proportion of activated plasmablasts however this requires further study.

The ELISA component of ALS for TB has typically used BCG vaccine as the coating antigen in order to incorporate a diverse range of antigenic epitopes from which to capture secreted antibodies [6, 31, 32]. Although BCG vaccine is widely administered throughout Tanzania, effects from prior vaccination on ALS responses are thought to be non-significant based on the methodology, which allows for antibody secretion during 48–72 hours of unstimulated PBMC culture. The lack of antigenic stimulation reduces the potential for ex-vivo influence from prior BCG vaccination or from latent TB disease states; the relatively short duration of cell culture minimizes the likelihood of detecting memory B cell responses [33, 34]. The use of other TB virulence factors as the coating antigen(s), including purified protein derivative (PPD), Antigen 85 complex, lipoarabinomannan (LAM), Mtb specific antigen (TB-SA) and those from the region of difference-1 (RD-1) domain of the Mtb genome has been evaluated [4, 6, 35, 36]. In the study by Rekha *et al* of 212 Bangladeshi adults undergoing evaluation for possible TB, the performance of ALS was compared after using various TB virulence factors singly or in combination to coat the ELISA plates. The best performance from a single additive was found with BCG (sensitivity of 90%, specificity of 88%), which is why we used this. The best performance from a combination of antigenic additives was found with BCG+LAM (sensitivity of 91%, specificity of 89%). Interestingly, the use of RD-1 proteins in isolation or combination had reduced performance (sensitivity of 73–86%, specificity of 83–86%) [6].

One of the limitations of our findings included the detection of inter-individual variation in ALS responses, which manifested as outliers in both of the disease groups. Regression analyses were used to mitigate any potential influence from these outliers, which demonstrated no overall effect on the associations. Possible explanations for the inter-individual differences in the magnitude of ALS responses could be from differences in the duration of infection, or potentially from strain-specific effects on immune activation. Different strains of Mtb including laboratory strains and clinical/circulating strains have been postulated to account for variable immune responses, although the exact effects on B-cell activation are understudied [37, 38]. A limitation of the current methodology is that it requires considerable laboratory infrastructure for harvesting and culturing PBMCs in real time which may preclude its use in resource limited areas. If further evaluation proves successful, it may be possible to incorporate an ELISpot platform for lateral flow analysis, as has been done in other settings for vaccine-induced immune responses [39].

Our study differs from other similar studies in that our threshold for assay positivity was found to be lower than previously published: 0.135 OD compared to 0.425 OD [4–6]. It is possible that these differences are due to variability in ELISA technique, or even different basal rates of anti-mycobacterial plasmablast activity among endemic “healthy controls” based on local TB exposure intensity. Indeed the absolute numbers of incident cases would suggest that Bangladesh and Ethiopia have higher TB transmission [1]. Additionally, the previously studied cohorts included patients who were found to have non-TB diagnoses as controls, including bacterial pneumonia, bronchiectasis, chronic obstructive pulmonary disease, and malignancy [4–6]. In our study, we used a case-control study design in which the controls included those without symptoms of infection or TB; the controls in this study were most commonly hospitalized with complications related to hypertension, diabetes, anemia and/or abdominal pain. This selection of controls may predispose to spectrum bias, which has the potential to overestimate the performance of the assay and affect the calculated thresholds. A cohort study design may address these issues and improve the generalizability of the findings.

In summary, we have demonstrated that incident B-cell responses to TB can be measured from peripheral blood and serve as a biomarker of TB disease activity among adults. ALS responses did not correlate with the bacillary burden of disease, as measured by sputum AFB smear status or HIV status, thereby conferring a unique niche for an immunologic biomarker for TB. Further evaluation of the assay seems warranted.

## Supporting Information

S1 Dataset Evaluation of ALS among TB cases and controls. (XLSX)
